# Performance of Different Combination Models of High-Risk HPV Genotyping in Triaging Chinese Women With Atypical Squamous Cells of Undetermined Significance

**DOI:** 10.3389/fonc.2019.00202

**Published:** 2019-04-03

**Authors:** Zhen Guo, Man-Man Jia, Qiong Chen, Hong-Min Chen, Pei-Pei Chen, Dong-Mei Zhao, Ling-Yan Ren, Xi-Bin Sun, Shao-Kai Zhang

**Affiliations:** ^1^Central Laboratory, Affiliated Cancer Hospital of Zhengzhou University, Henan Cancer Hospital, Zhengzhou, China; ^2^Department of Gynecological Oncology, Affiliated Cancer Hospital of Zhengzhou University, Henan Cancer Hospital, Zhengzhou, China; ^3^Department of Cancer Epidemiology, Affiliated Cancer Hospital of Zhengzhou University, Henan Cancer Hospital, Zhengzhou, China; ^4^Department of Pathology, Affiliated Cancer Hospital of Zhengzhou University, Henan Cancer Hospital, Zhengzhou, China

**Keywords:** human papilloma virus (HPV), genotyping, triage, atypical squamous cells of undetermined significance (ASCUS), cervical cancer

## Abstract

**Objective:** The purpose of this study was to evaluate the effect of different combination models of high-risk human papilloma viruses (HPV) genotyping in triaging Chinese women with atypical squamous cells of undetermined significance (ASCUS).

**Methods:** We established a screening cohort of 3,997 Chinese women who underwent cervical cytology and HPV genotyping test. Women with ASCUS cytology underwent punch biopsy under colposcopy/endocervical curettage. The sensitivity, specificity, positive predictive value (PPV), and negative predictive value (NPV) of different combination models of HR-HPV genotyping calculated that cervical intraepithelial neoplasia 2 or higher (CIN2+) on histology were endpoints.

**Results:** Of the full sample, 393 women had ASCUS. Among ASCUS women with a CIN2 lesion, the prevalence for HPV were 40.0% (type 16), 10.0% (type 18), 0.0% (type 33), 30.0% (type 52), 40.0% (type 58), and 30.0% (other nine types). For ASCUS women with a CIN3 lesion, the prevalence for HPV were 68.4% (type 16), 15.8% (type 18), 10.5% (type 33), 31.6% (type 52), 15.8% (type 58), and 36.8% (other nine types). Combination model including HPV16/18/33/52/58 for predicting CIN2+ lesion in women with ASCUS had relatively higher sensitivity [93.1% (78.0, 98.1)], specificity [75.8% (71.2, 79.9)], PPV [23.5% (16.7, 32.0)], and NPV [99.3% (97.4, 99.8)] than other combination models. Moreover, the referral rate of HPV16/18/33/52/58 (29.3%) was lower than HR-HPV (36.1%).

**Conclusions:** The study demonstrates that specific HR-HPV types HPV16/18/33/52/58 may be an effective strategy in ASCUS triage. This improves the subsequent selection of ASCUS patients.

## Introduction

Cervical cancer is the third most common cancer among women worldwide, the second most common cause of cancer death, and causes 300,000 deaths a year (1). Nevertheless, of all malignant tumors, cervical cancer is the one that is most easily preventable by screening (2). Therefore, selecting effective screening methods for cervical precancerous lesions is especially important. The current suggestion for diagnosing cervical lesions involve a “three-step” exanimation: liquid based cytology, colposcopy (which is an observation technique that can identify potential precancerous and cancerous lesions), and histological examination (3). Women may require further follow-up or treatment, or both, depending on the severity of the lesion if cytological atypia are present. Women with high-grade cytological lesions should be referred immediately for further examination using the reference standard test that involves colposcopy and histological examination of colposcopy-targeted biopsies (4, 5). However, the management of women with atypical squamous cells of undetermined significance (ASCUS) remains controversial (6). An ASCUS result is not a true biological entity that progresses or regresses, but rather one which represents an equivocal diagnosis from normal, lower-grade squamous intraepithelial lesions (LSIL), high squamous intraepithelial lesions (HSIL) to invasive cancer (7). The 2006 consensus guidelines for the management of women with abnormal cytological smears recommend three approaches for the management of ASCUS: 2 repeat cervical smears taken 6 months apart, reflex HR-HPV DNA testing, and colposcopic examination (8). These are all safe and effective choices and the approach taken depends on the individual circumstances and resources available.

Most women with ASCUS do not have clinically significant diseases, nonetheless, a substantial proportion (15–20%) of them do have cervical intraepithelial neoplasia (CIN) in a follow-up histopathological diagnosis (9). Research has shown that the 5-year risk of CIN3 or higher (CIN3+) in ASCUS women was significantly higher than that of the general population (10). Hence, the accurate triage of ASCUS women is required to identify those who really need further management.

Persistent infection of high-risk (HR) genotypes of human papillomavirus (HPV) may lead to the development and progression of cervical cancer (11, 12). In the past 10 years, an HPV test has been used to guide the management for ASCUS women by recommending only those women with positive HR-HPV for diagnostic colposcopy (13). However, the roles of HPV in the development of CIN and invasive cancer differ depending on the HPV genotype, as the carcinogenicity of the different HPV types differs. HPV genotypes 16, 18, 31, 33, 35, 39, 45, 51, 52, 56, 58, and 59 are carcinogenic; HPV68 is probably carcinogenic; and HPV 26, 30, 34, 53, 66, 67, 69, 70, 73, 82, 85, and 97 are possibly carcinogenic (14). HPV16 and/or HPV18 infection is found in 52% of CIN cases and 70% of all cervical cancer cases (15). In women with HPV infection and an initially normal cytology, the rates of developing CIN2 or higher (CIN2+) in 13.4 years of follow-up were reported to be 28.5% in women with HPV16 alone, 15.4% in women with HPV18 alone, and 19.1% (type 33), 18.2% (type 35), 16.7% (type 58), 15.7% (type 31), 8.6% (type 51), 8.5% (type 45), 4.7% (type 52), 3.6% (type 39), or 2.8% (type 56) HPV positive women (16). These data provide a rationale for selecting appropriate HPV genotypes in triaging ASCUS cases.

The ideal triage strategy of HPV genotyping for ASCUS cases is one where sensitivity and specificity can be maximized, which can reduce the rate of missed diagnosis and avoid unnecessary referral for colposcopy. The present study was designed to evaluate the effectiveness of HR-HPV genotype testing for triaging Chinese women with ASCUS, and to provide a new parameter for formulating the best triage strategy of ASCUS.

## Methods

### Sample Inclusion Criteria

A cervical cancer screening cohort of 3,997 women was established in Jiyuan City, Henan from April to May 2017. Participants underwent cervical cytology and HPV genotype testing. Women were eligible for the study if they were >21 years of age; had sex; were not currently pregnant; had no history of hysterectomy, cervix surgery, or cervical cancer treatment; and were able to provide informed consent. This study was approved by the Institutional Review Board of Affiliated Cancer hospital of Zhengzhou University.

### Screening Procedures

Prior to study enrollment, a trained health care worker obtained informed consent and administered a questionnaire in a confidential interview with each eligible woman to assess medical and surgical history of the cervix uteri and cervical cancer; marital status; educational levels; smoking; drinking history; and reproductive information.

Each eligible woman had a gynecological examination of their vulva, vagina, and uterine neck. A physician performed speculum exams, collected specimens of cervical exfoliated cells by broom brush, and transferred the cells to a PreservCyt liquid (Hologic Inc., Boston, USA), stored at 4°C for liquid-based cytology classification and HPV DNA testing.

**Figure 1:**
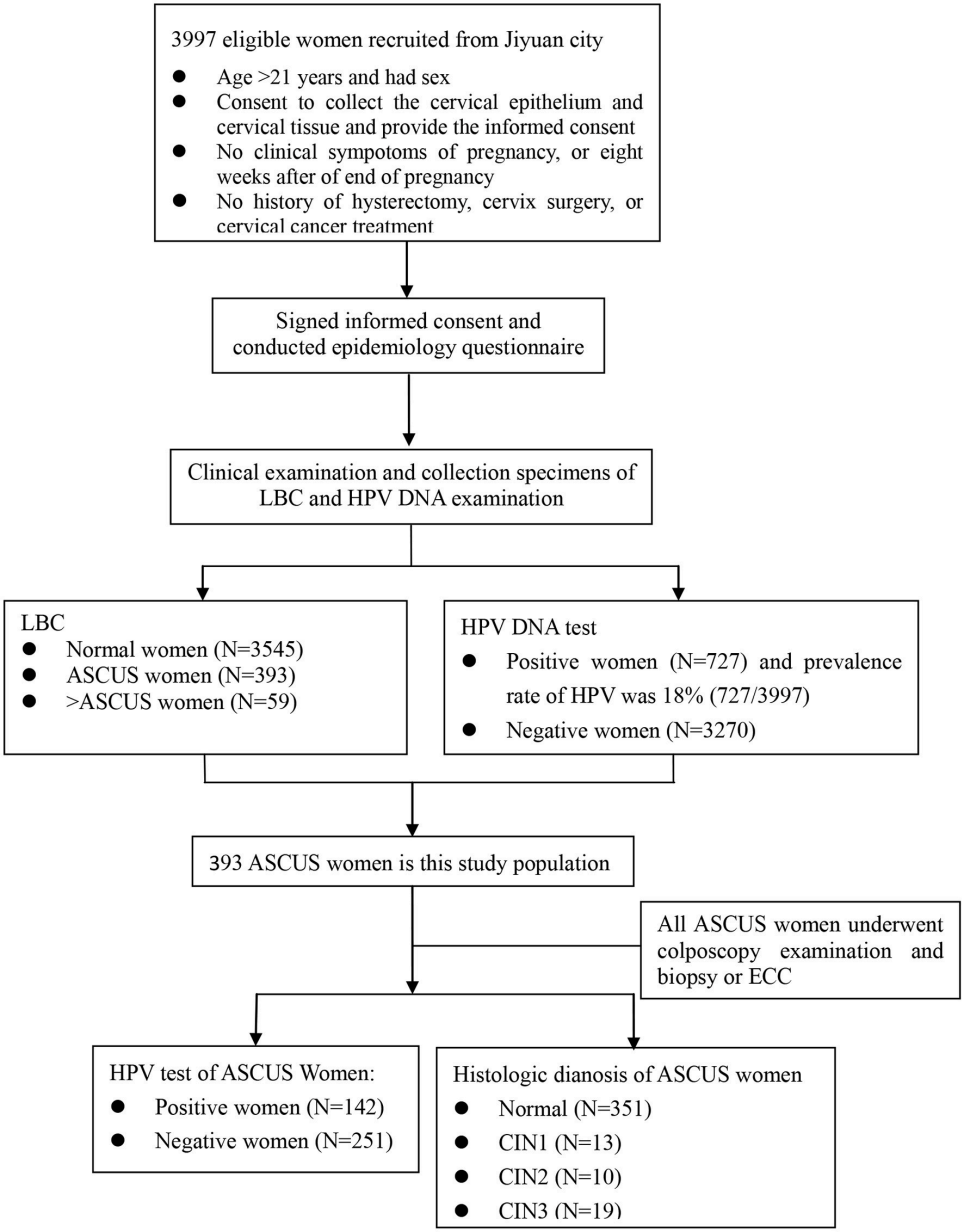
The flowchart of this study. LBC, liquid-based cytology; ASCUS, Atypical squamous cells-undetermined significance; ECC, Endocervical Curettage; CIN1/2/3, Cervical intraepithelial neoplasia grade 1/2/3.

Liquid-based cytology (LBC) testing results were ASCUS women who had been required recall for colposcopy examination with 12 weeks after collecting specimens of cervical exfoliated cells. An experienced gynecologist performed colposcopy-directed biopsies on women with ASCUS. If the squamo-columnar junction was completely visible, it was a satisfactory colposcopy examination. Women with normal colposcopy examination were not needed to undergo biopsies. Conversely, women with abnormal colposcopy examination underwent directed cervical biopsies where lesions were visible. If the colposcopy examination was unsatisfactory (the squamo-columnar junction was not completely visible), physicians performed endocervical curettage (ECC) (Figure 1).

### HPV Testing

We performed HPV genotyping using an HPV DNA Genotyping Kit (Tellgen Cor., Shanghai, China) according to the manufacturer's instructions. The HR-HPV test was based on multiple nucleic acid amplification Polymerase Chain Reaction (PCR) method with fluorescence detection. Primers and probes were designed specifically for target sequences of the L1 gene for high-risk HPV. PCR procedure could detect 14 types of HPV DNA (16, 18, 31, 33, 35, 39, 45, 51, 52, 56, 58, 59, 66, and 68) from the cervical exfoliative cells and distinguish HPV types 16, 18, 33, 52, and 58. Each experiment had positive and negative quality control and blank control. We synchronously measured reference gene β-Globin for judging a false negative either due to insufficient sampling or operation error.

### Cytology and Histology

We used the Thinprep liquid-based cytologic test (TCT). The Bethesda system (TBS) was used for cytology and the CIN classification systems for histology. The worst reading across all histological findings was the final diagnosis for each woman. A woman was assessed as negative for CIN if a biopsy had not been indicated or the histology finding was negative. Cyto-pathologists and pathologists from the Zhengzhou University Affiliated Cancer Hospital prepared and read the cytological and histological slides. Before the study began, the Hospital held a training meeting on colposcopy and histopathology diagnosis to standardize the protocol.

### Statistical Analysis

We analyzed the data using SPSS version 20.2 (IBM Corp., New York, USA). All women with ASCUS underwent colposcopy and/or ECC. The referral rate was calculated as number of ASCUS patients with positive HR-HPV type divided by the total number of ASCUS patients. We estimated the mean and standard deviation of the quantitative variables and numbers and percentages for categorical variables. Sensitivity, specificity, positive predictive value (PPV), and negative predictive value (NPV) of HPV genotyping for detecting cervical lesions CIN2+ were calculated and compared to the pathological diagnosis as the gold standard. From these results, we could evaluate the accuracy and effectiveness of HPV genotyping in triaging women with ASCUS. *P* ≤ 0.05 (two-sided) were considered statistically significant.

## Results

Of the overall cervical cancer screening cohort (3,997), a total of 393 (9.8%) women had cytologically confirmed ASCUS. Table 1 presents the characteristics of the ASCUS sample. Age was described by mean. The mean age of ASCUS women was 50.8 ± 9.2 years (range, 21–64 years). The mean age of menarche was 15.2 ± 1.7 years and the mean age of menopause was 46.3 ± 6.8 years. About 80% of women completed primary or junior school education. All of the women had never smoked. 99.2% women had never drunk. About 27.2% of women had more than 3 pregnancies and 7.9% had more than 3 reproductions.

**Table 1:** Characteristics of the study population.

**Characteristics**	***N***	**Mean ($\left(\bar{x}\pm s\right)$) or prevalence (%)**
**Age**	393	50.8 ± 9.2
Age of menarche	393	15.2 ± 1.7
Age of menopause	393	46.3 ± 6.8
Age at first pregnancy	393	22.6 ± 3.0
Age at first birth	393	23.4 ± 3.1
**Level of education**		
Uneducated	37	9.4
Primary education	137	34.9
Junior middle school education	167	42.5
High school education	44	11.2
≥University education	8	2.0
**Smoking**		
Yes	0	0.0
No	393	100
**Drinking**		
Yes	3	0.8
No	390	99.2
**Times of pregnancy**		
≤3	286	72.8
>3	107	27.2
**Times of reproduction**		
≤3	362	92.1
>3	31	7.9
**HPV infection**		
Positive	142	36.1
Negative	251	63.9

The prevalence of the screening cohort was 18.2% (727/3,997) (Table S1). Table 2 shows the prevalence of different HPV genotypes in women with ASCUS. The prevalence of HPV types in this study were 48.5% for HR-HPV, 29.3% for HPV16/18/33/52/58, and 13.0% for HPV16/18. The prevalence of HR-HPV increased with the degree of severity of pathological diagnosis, which was 31.1% in women with normal pathology results and 100% in women with CIN3. Among ASCUS women with CIN2, the prevalence were 40.0% for HPV type 16, 10.0% for type 18, 0.0% for type 33, 30.0% for type 52, 40.0% for type 58, and 30.0% for the other nine types. In women with CIN3, the prevalence were 68.4% (type 16), 15.8% (type 18), 10.5% (type 33), 31.6% (type 52), 15.8% (type 58), and 36.8% (other nine types) (Table 3).

**Table 2:** Prevalence of different HPV genotypes model in women with ASCUS.

**Triage criteria using HR-HPV types**	**Positive**		**Negative**	
	***n***	**%**	***n***	**%**
HPV16/18	51	13.0	342	87.0
HPV16/18/52	58	14.8	335	85.2
HPV16/18/52/58	86	21.9	307	78.1
HPV16/18/33/52/58	115	29.3	278	70.7
HR-HPV^*^	142	36.1	251	63.9

*HPV, human papillomavirus; HPV16/18, HPV16 and/or HPV 18 is positive; HPV16/18/52, either of HPV 16, 18, 52 is positive; HPV16/18/52/58, either of HPV 16, 18, 52, 58 is positive; HPV16/18/33/52/58, either of HPV 16, 18, 33, 52, 58 is positive; HR-HPV, high risk-human papillomavirus*;

* *including HPV types 16, 18, 31, 33, 35, 39, 45, 51, 52, 56, 58, 59, 66, 68*.

**Table 3:** Distribution of HPV type according to histologic diagnosis.

**Histologic diagnosis**	***N***	**HPV 16**	**HPV 18**	**HPV 33**	**HPV 52**	**HPV 58**	**Other nine**	HR-HPV^*^
		***n* (%)**	***n* (%)**	***n* (%)**	***n* (%)**	***n* (%)**	***n* (%)**	***n* (%)**
Total	393	43 (11.0)	12 (3.1)	10 (2.5)	46 (11.7)	36 (9.2)	74 (18.8)	142 (36.1)
Normal	351	24 (6.8)	7 (2.0)	7 (2.0)	36 (10.3)	29 (8.3)	61 (17.4)	109 (31.1)
CIN1	13	2 (15.4)	1 (7.7)	1 (7.7)	1 (7.7)	0 (0.0)	3 (23.1)	5 (38.5)
CIN2	10	4 (40.0)	1 (10.0)	0 (0.0)	3 (30.0)	4 (40.0)	3 (30.0)	9 (90.0)
CIN3	19	13 (68.4)	3 (15.8)	2 (10.5)	6 (31.6)	3 (15.8)	7 (36.8)	19 (100.0)

*HPV, human papillomavirus; CIN, cervical intraepithelial neoplasia; CIN2+, cervical intraepithelial neoplasia grade 2 or worse; HR-HPV, high risk-human papillomavirus, other nine including HPV types 31, 35, 39, 45, 51, 56, 59, 66, 68*;

* *including HPV types 16, 18, 31, 33, 35, 39, 45, 51, 52, 56, 58, 59, 66, 68*.

Cervical lesions CIN2+on histology was as the endpoint, the sensitivity and NPV of different combination models of HR-HPV increased with HPV16/18, HPV16/18/52, HPV16/18/52/58, HPV16/18/33/52/58, and HR-HPV (Table 4). However, the specificity and PPV decreased as the number of HPV genotype combinations increased. HPV 16/18 has the highest specificity for triaging patients with ASCUS (91.2%), but it also had the lowest sensitivity (65.5%) (Table 4). For HR-HPV, the sensitivity was 96.6% (82.8, 99.4), specificity was 68.7% (63.7, 73.2), PPV was 19.7% (14.0, 27.0), and NPV was 99.6% (97.8, 99.9). The sensitivity and NPV of HPV16/18/33/52/58 were similar to HR-HPV. However, the specificity and PPV of HPV16/18/33/52/58 for detecting CIN2+ in women with ASCUS were both higher figures than HR-HPV. Moreover, the referral rate for HPV16/18/33/52/58 (29.3%) was lower than HR-HPV (36.1%) (Table 4).

**Table 4:** The effect of HPV genotyping testing in triaging women with ASCUS.

**Triage criteria using HR-HPV types**	**Sensitivity**		**Specificity**		**PPV**		**NPV**		**Referral rate (%)**
	**%(*n*/*N*)**	**95%CI**	**%(*n*/*N*)**	**95%CI**	**%(*n*/*N*)**	**95%CI**	**%(*n*/*N*)**	**95%CI**	
**CIN2+**									
HPV16/18	65.5 (19/29)	47.3, 80.1	91.2 (332/364)	87.9, 93.7	37.3 (19/51)	25.3, 51.0	97.1 (332/342)	94.7, 98.4	13.0
HPV16/18/52	79.3 (23/29)	61.6, 90.2	82.4 (300/364)	78.2, 86.0	26.4 (23/87)	18.3, 36.6	98.0 (300/306)	95.8, 99.1	14.8
HPV16/18/52/58	89.7 (26/29)	73.6, 96.4	77.2 (281/364)	72.6, 81.2	23.9 (26/109)	16.8, 32.7	99.0 (282/285)	97.0, 99.6	21.9
HPV16/18/33/52/58	93.1 (27/29)	78.04, 98.09	75.8 (276/364)	71.2, 79.9	23.5 (27/115)	16.7, 32.0	99.3 (277/279)	97.4, 99.8	29.3
HR-HPV	96.6 (28/29)	82.8, 99.4	68.7 (250/364)	63.7, 73.2	19.7 (28/142)	14.0, 27.0	99.6 (250/364)	97.8, 99.9	36.1

*CIN2+, cervical intraepithelial neoplasia grade 2 or worse; 95% CI, 95% confidence interval; PPV, positive predictive value; NPV, negative predictive value; ASCUS, atypical squamous cells of undetermined significance*.

## Discussion

An ASCUS result is the most common non-normal cytologic finding in cervical cancer screening, which is either an actively proliferated benign lesion or a potentially malignant lesion; the histopathology results of which are very different. Therefore, the establishment of management standards for patients with ASCUS is urgently needed. In recent years, HPV DNA testing has been incorporated into screening programs, which is firstly a means of triaging patients with ASCUS (17, 18), and subsequently as part of co-testing with a TCT test. Each HR-HPV type predisposes patients to a different risk of developing CIN and invasive cancer. The efficacy of genotyping specific HR-HPV types in triaging ASCUS cases may differ according to the specific combination of HPV types tested. Therefore, it is necessary to choose a combination model of HPV types with high sensitivity and specificity as well as lower referral rates for the triage of ASCUS. Most previous studies only evaluated the role of HPV16/18 or HR-HPV in triaging abnormal cervical cytology. Lin et al. 's study evaluated the effect of HPV16/18 and other HR-HPV types in the triage of ASCUS and LSIL (19). Jiang et al. 's study analyzed the role of 10 HR-HPV types in the triage 25–36 years old younger women with abnormal cytology (20). However, our study assessed the effect of different combinations models of HR-HPV genotyping (HPV16/18, HPV16/18/52, HPV16/18/52/58, HPV16/18/33/52/58, 14 HR-HPV) in triaging Chinese women with ASCUS by cytology. We found that a model of HPV16/18/33/52/58 had relatively higher sensitivity, specificity, PPV and NPV, as well as lower referral rate than other models of HPV types in triaging ASCUS cases.

In the previous studies, the percentage of ASCUS in cervical cytology was found to range between 3 and 10% (21–23). Similarly, our study found that the ratio of ASCUS was 10%. The prevalence of HR-HPV in ASCUS in different studies were significantly different. One study showed that the prevalence of HR-HPV in ASCUS was found to be as high as 41% (24). While the rate was 18% in another study (25). The positive rate of HR-HPV in women with ASCUS was 49% in our study. In addition, we found that the prevalence of HPV 16, HPV18, HPV 33, HPV 52, HPV 58 in ASCUS patient samples with CIN3 lesion was 68, 16, 11, 32, and 16%, respectively. These findings indicate that women with ASCUS and HPV 16, 18, 33, 52, or 58 infections may harbor high-grade CIN, demonstrating the importance of developing specific HPV genotyping tests.

A meta-analysis comparing the accuracy of HR-HPV testing against that of repeated cytology for detection of underlying CIN2+ or CIN3+ in women with ASCUS showed that HR-HPV-triage had significantly higher sensitivity, but not significant specificity than repeated cytology in ASCUS triage (2). In our study, the sensitivity of HR-HPV detecting CIN2+ in women with ASCUS was 97%, but specificity was only 69%. Using the HR-HPV to triage women with ASCUS will increase the referral rate and create unnecessary cost and resource burdens for patients. A study indicated HPV types 16, 33, 58, 51, and 52 were the main HPV genotype in a rural Chinese population, accounting for 88% of all HPV infections (26). Our results were in a general agreement with the above-mentioned study, which showed that HPV 16, 18, 33, 52, and 58 had relatively higher infection rates in ASCUS cases with CNI2 or CIN3 lesion. Hence, we evaluated the sensitivity, specificity, PPV and NPV using different HPV type combinations in our models, which included HPV16/18, HPV16/18/52, HPV16/18/52/58, HPV16/18/33/52/58, and HR-HPV for detecting underlying CIN2+ in women with ASCUS. Like previous studies (27), the results showed that combination of HPV16/18 as a means to predict CIN2+ in ASCUS cases increased the specificity compared with HPV16/18/33/52/58 (91% vs. 76%), but it showed significant decreases in sensitivity (66% vs. 93%) which resulting in relatively higher rate of missed diagnosis. However, we found higher sensitivity of HPV16/18/33/52/58 in detecting CIN2+ in ASCUS cases compared with HPV16/18/52, HPV16/18/52/58 (93% vs. 80%, 93% vs. 89%), and the specificity of HPV16/18/33/52/58 was higher than HR-HPV (76% vs. 67%). Moreover, ASCUS patients with specific HR-HPV type are often suggested to referral for colposcopy, while the referral rate of HPV16/18/33/52/58 was significantly lower than that of HR-HPV (29% vs. 36.1%). Therefore, we consider the HPV16/18/33/52/58 genotypes to be an adequate triage strategy for the detection of CIN2 or higher in patients with ASCUS in China.

This study has several limitations. First, we did not test and classify all HPV genotypes, so every combination of all HPV genotypes can't be assessed. However, the infection rates of HPV16/18/33/52/58 are highest in Chinese women and this study showed the model with these types was best at triaging ASCUS women. Second, this study was conducted in a single region, which may reduce generalizability to other regions. We did not perform external validation in additional datasets. In addition, this study lacked a significant follow up in our cases. As a result, the study population may not be reflective of a true screening population.

In summary, this is the first study in China to assess the effect of different models of HR-HPV genotyping in triaging Chinese women with ASCUS cytology. Our study demonstrates that genotyping for HPV16/18/33/52/58 genotypes serves as a robust triage system for ASCUS women based on its high sensitivity, specificity, and the minimal number of HR-HPVs needed for genotyping. The development of a specific HPV genotyping assay may significantly improve the cost-effectiveness of screening. In the next step, we plan to externally validate our novel HPV genotyping assay with a different dataset.

## Data Availability

All datasets generated for this study are included in the manuscript and/or the Supplementary Files.

## Ethics Statement

This study was approved by the Institutional Review Board of Affiliated Cancer Hospital of Zhengzhou University. Each participant was informed and signed an informed consent.

## Author Contributions

ZG and S-KZ contributed to the design and write wrote the manuscript. ZG, QC, and P-PC performed statistical analysis. ZG, M-MJ, and P-PC contributed to investigation and HPV test. H-MC, D-MZ, and L-YR contributed to cytology and histology examination. X-BS helped conceiving the study and assisted with the statistical analyses. All authors read and approved the final manuscript.

### Conflict of Interest Statement

The authors declare that the research was conducted in the absence of any commercial or financial relationships that could be construed as a potential conflict of interest.

## Abbreviations

HPV: Human Papilloma Virus
ASCUS: Atypical squamous cells of undetermined significance
PPV: Positive predictive value
NPV: Negative predictive value
CIN: Cervical intraepithelial neoplasia
HR: High risk
LSIL: Lower-grade squamous intraepithelial lesions
ECC: Endocervical curettage
PCR: Polymerase Chain Reaction
TCT: Thinprep cytologic test
TBS: The Bethesda system
CI: Confidence intervals.
