# Supplementary material for: Performance of Different Combination Models of High-Risk HPV Genotyping in Triaging Chinese Women With Atypical Squamous Cells of Undetermined Significance
Source: Front Oncol. 2019 Apr 3;9:202. doi: 10.3389/fonc.2019.00202 (PMC6456653; doi:10.3389/fonc.2019.00202)
Supplement: Supplementary file 1 [file Table_1.docx]

Table S1 Distribution HPV type Among 3977 women of screening cohort

| HPV type | N | Positive (%) |  | Negative(%) |
| --- | --- | --- | --- | --- |
| HPV16 | 3997 | 141(3.5) |  | 3856(96.5) |
| HPV18 | 3997 | 42(1.1) |  | 3955(98.9) |
| HPV33 | 3997 | 56(1.4) |  | 3941(98.6) |
| HPV52 | 3997 | 181(4.5) |  | 3816(95.5) |
| HPV 58 | 3997 | 135(3.4) |  | 3862(96.6) |
| Other nine | 3997 | 392(9.8) |  | 3605(90.2) |
| HR-HPV* | 3997 | 727(18.2) |  | 3270(81.8) |

HPV, human papillomavirus; HR-HPV, high risk-human papillomavirus, other nine including HPV types

31, 35, 39, 45, 51, 56, 59, 66, 68; *including HPV types 16, 18, 31, 33, 35, 39, 45, 51, 52, 56, 58, 59, 66, 68.
